# Use of coupled ion mobility spectrometry-time of flight mass spectrometry to analyze saturated and unsaturated phenylpropanoic acids and chalcones

**DOI:** 10.1186/1752-153X-8-38

**Published:** 2014-06-21

**Authors:** Mwafaq Ibdah, David R Gang

**Affiliations:** 1 Institute of Biological Chemistry, Washington State University, PO Box 646340, Pullman, WA 99164-6340, USA; 2 Agriculture Research Organization, Institute of Vegetable and Field Crops, Newe Ya’ar Research Center, P.O. Box 1021, Ramat Yishay 30095, Israel

**Keywords:** Ion mobility spectrometry, Mass spectrometry, Phenylpropanoic acids, Flavonoids, Chalcone, Dihydrochalcone

## Abstract

**Background:**

In metabolite profiling screens or analyses, where generic separation and analysis conditions are used in efforts to measure as many metabolites as possible, overlapping signals from very similar molecules often make it very difficult if not impossible to separate and identify specific molecules of specific classes. The aim of this study was to evaluate the utility of coupling ion mobility spectrometry to UPLC-TOFMS (UPLC-Q-IMS-TOFMS) as a means to separate and identify saturated and unsaturated phenylpropanoic acids and chalcones, phenylpropanoid-acetate pathway derived compounds that are common in plant extracts.

**Results:**

This approach readily separated most of the unsaturated phenylpropanoid acids (*t*-cinnamate, *p*-coumarate, caffeate, ferulate) from the corresponding saturated (dihydro-) compounds, and analysis of two dimensional plots of mass/charge ratio values versus ion mobility drift time revealed that the other compounds can indeed be distinguished. However, this approach was less effective for the larger chalcones.

**Conclusions:**

UPLC-Q-IMS-TOFMS is a promising tool to enable the separation, identification and quantification of very similar molecules. Although it has its limitations, as was seen for the chalcones that were not well separated in this investigation, ion mobility spectrometry nevertheless adds an additional level of characterization to large-scale metabolomic screens, which increases the power of such screens without the demand for multiple analyses using very different column chemistries.

## Background

The natural dihydrophenylpropanoid metabolites, such as dihydroferulic acid, dihydrosinapic acid, and dihydrocaffeic acid, have been identified in several plants and accumulate as heartwood-forming constituents [1,2]. These dihydrophenylpropanoids, which contribute to the color, quality, and durability of the woody tissues [2], have either propanol, propionic acid or propanaldehyde side chains, e.g.*, p*-dihydrocoumaric acid, dihydroferulic acid, *p*-dihydrocoumaroyl alcohol and dihydroconiferyl alcohols. These compounds are of interest to human health because of their multiple biological and pharmacological (antioxidant and radical scavenging) properties [3,4]. They differ from their ubiquitous unsaturated counterparts, which are intermediates in the general phenylpropanoid pathway and are similarly widespread in plants, by possessing saturated propanyl side chains.

Chalcones and dihydrochalcones belong to another important class of plant phenolics, the flavonoids. Chalcones are defined by the presence of two aromatic rings linked by a three-carbon bridge that is unsaturated in chalcones and saturated in dihydrochalcones. Chalcones and dihydrochalcones have been found to have a number of important biological activities [5], including anti-inflammatory [6], antioxidant [7], antiplasmodial [8], anticholinesterase [9] and anticancer properties [10,11], and thus development of means to better detect, identify and measure the levels of such compounds in biological samples is of great importance.

The identification and analysis of such compounds in extracts from complex plant tissues is of great interest for metabolomic analyses and non-targeted phytochemical screens due to their ubiquity in the plant kingdom. However, the precise analysis of phenylpropanoids and flavonoids in plant tissues can be a complex task because of the variety of similar structures present in such molecule classes [12]. As a result, such compounds often co-elute in typical metabolomic or metabolite profiling experiments. Because mass spectrometric analysis is unable to distinguish between isobaric substances and structural information is often lacking due to the absence of standard compounds, which would enable MS/MS analyses that could lead to better identification and quantification, it is often impossible without an orthogonal analysis technique to identify and quantify specific compounds in these classes, unless complex chromatographic separations utilizing multiple column chemistries is employed, which is undesirable in metabolomic-scale investigations.

Ion mobility spectrometry (IMS) coupled inline to UPLC-TOFMS (UPLC-Q-IMS-TOFMS) is a promising and powerful analytical tool for the analysis of the diversity of saturated and unsaturated phenylpropanoid-acetate pathway derived compounds in plant extracts [13,14]. In the present study, the utility of UPLC-Q-IMS-TOFMS (using a Waters SYNAPT G2 HDMS instrument) was evaluated for its ability to analyze a series of saturated and unsaturated phenylpropanoic acids and simple chalcones.

## Results and discussion

An Acquity UPLC BEH C18 column was selected for this work due to the separation efficiency, peak shape and signal intensity of the 12 tested compounds, as well as its common use in metabolomic investigations. The combined mixture, consisting of the saturated and unsaturated phenylpropanoic acids and chalcones, eluted within 6 min. In addition to MS analysis, compound elution was monitored by the diode array (220 to 490 nm), which showed a broad peak of non-separated compounds for all compound pairs (saturated vs. unsaturated forms). Attempts to optimize compound separation failed to provide baseline separation in the UV chromatograms, using either the BEH C18 or a number of other columns such as CSH (Charged Surface Hybrid) and HSS (High Strength Silica).

Because of the difference in mass between the saturated and unsaturated compounds, and because high resolution MS (i.e., >10,000 full width at half maximum, FWHM) was used, it was possible to differentiate specific compounds in selected ion chromatograms, when standard compounds were available for comparison. However, in the absence of a chemical standard as reference, such as would be expected in *bona fide* metabolite identification scenarios when metabolites would be analyzed directly from a biological matrix, it would be difficult to identify many of these metabolites based on MS chromatograms and corresponding MS and/or MS/MS spectra alone. Although the application of very high resolution and high mass accuracy mass spectrometry would assist in this characterization, being unable to distinguish regio-isomers, such an approach is still viewed as being inadequate to be able to characterize the compounds of interest in many instances, especially in complex biological samples. To enable better separation and compound verification, IMS was added to the analysis. The drift times in the inline traveling-wave ion mobility spectrometer (TWIMS) were corrected for the mass-dependent flight time, defined as the time that an ion spent in the TOF analyzer [15].

### Analysis of saturated and unsaturated phenylpropanoic acids

**Figure 1:**
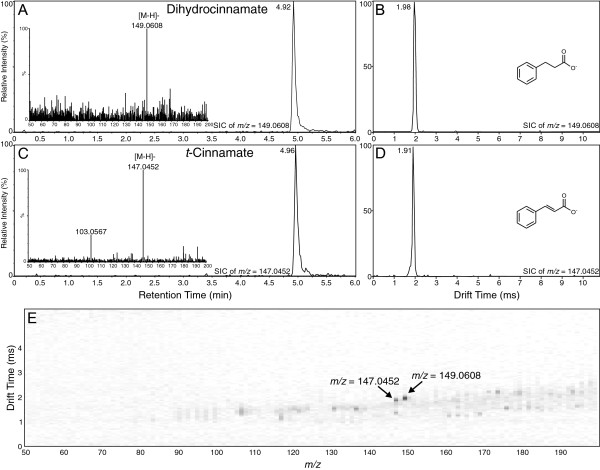
**Analysis of dihydrocinnamate and *trans*-cinnamate by UPLC-Q-IMS-TOFMS. A)** Selected ion UPLC-MS chromatogram of dihydrocinnamate ion ([M-H]^-^*m/z* = 149.0608; calculated = 149.0602, -4.03 ppm error) with MS/MS spectrum inset. **B)** Ion mobility spectrum of dihydrocinnamate ion ([M-H]^-^). **C)** Selected ion UPLC-MS chromatogram of *t*-cinnamate ion ([M-H]^-^*m/z* = 147.0452; calculated = 147.0446, -4.08 ppm error) with MS/MS spectrum inset. **D)** Ion mobility spectrum of *t*-cinnamate ion ([M-H]^-^). **E)** 2-D plot of drift time vs. *m/z* for combined mixture of dihydrocinnamate and *t*-cinnamate. Arrows indicate position of the two compounds. Image resolution was set to 1000 bins in the MS dimension to allow for ease of viewing of the ions.

The compounds investigated that were the most simple in terms of structure and functional groups were dihydrocinnamate and *trans*-cinnamate. As shown in Figure 1, these two compounds overlapped significantly in the UPLC analysis (Figure 1A and C), with retention times at 4.96 and 4.92 min, respectively. Baseline separation of these compounds was not possible under the UPLC conditions tested. However, when IMS was applied to the analysis of this compound pair, the unsaturated compound, *t*-cinnamate displayed a higher mobility (i.e., lower drift time) compared to the saturated dihydrocinnamate, at 1.91 ms versus 1.98 ms, respectively, (Figure 1B and D). This difference can be explained by the fact that the double bond of the unsaturated phenylpropanoic acids causes those compounds to be more rigid, and thus potentially more compact, compared to the saturated phenylpropanoic acids, leading to faster movement in the TWIMS, as can be readily seen in the 2-D plot generated by Driftscope (the software used to analyze IMS data in the SYNAPT G2 HDMS system) of drift time versus *m/z* for the combined mixture (Figure 1E).

**Figure 2:**
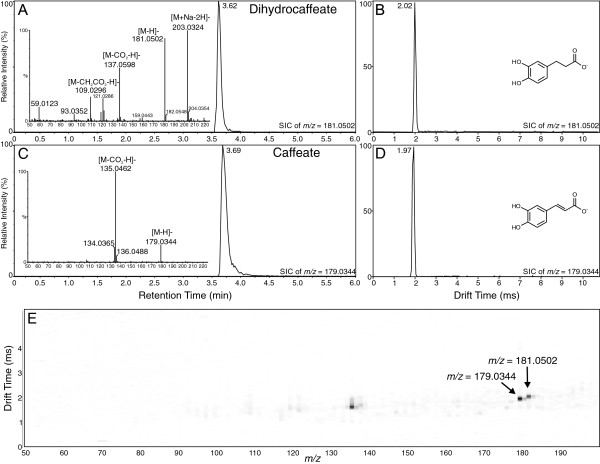
**Analysis of dihydrocaffeate and caffeate by UPLC-Q-IMS-TOFMS. A)** Selected ion UPLC-MS chromatogram of dihydrocaffeate ion ([M-H]^-^*m/z* = 181.0502; calculated = 181.0501, -0.55 ppm error) with MS/MS spectrum inset. **B)** Ion mobility spectrum of dihydrocaffeate ion ([M-H]^-^). **C)** Selected ion UPLC-MS chromatogram of caffeate ion ([M-H]^-^*m/z* = 179.0344; calculated = 179.0344, 0 ppm error) with MS/MS spectrum inset. **D)** Ion mobility spectrum of caffeate ion ([M-H]^-^). **E)** 2-D plot of drift time vs. *m/z* for combined mixture of dihydrocaffeate and caffeate. Arrows indicate position of the two compounds. Image resolution was set to 1000 bins in the MS dimension to allow for ease of viewing of the ions.

The same situation was observed when caffeate ([M-H]^-^*m/z* 179.0344) and dihydrocaffeate ([M-H]^-^*m/z* 181.0502), with addition of two hydroxyl groups at the *meta*- and *para*- positions compared to the *t*-cinnamate/dihydrocinnamate analogs, were analyzed (see Figure 2). The elution profiles of these two compounds from the UPLC overlapped significantly (Figure 2A and C), as did their drift times, being 1.97 ms and 2.02 ms, respectively (Figure 2B and D). Thus, analysis by either UPLC-MS or UPLC-IMS alone was not able to separate these compounds. However, as shown in Figure 2E, when UPLC-MS and IMS were applied together in the UPLC-Q-IMS-TOFMS system, as shown in the 2-D plot generated by Driftscope of drift time versus *m/z* for the combined mixture, clear separation of the two compounds is visible, as was seen for the *t*-cinnamate/dihydrocinnamate pair (see Figure 1E).

**Figure 3:**
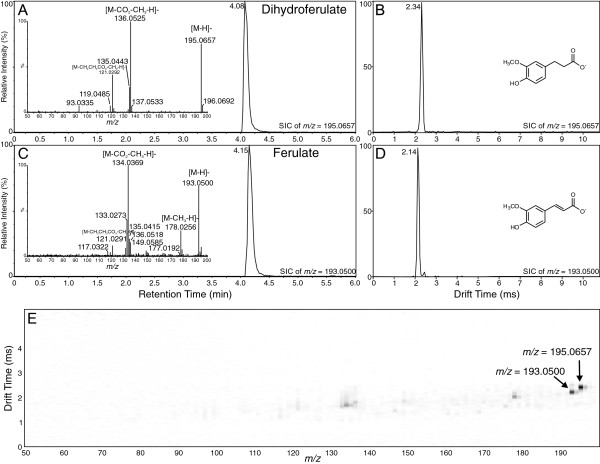
**Analysis of dihydroferulate and ferulate by UPLC-Q-IMS-TOFMS. A)** Selected ion UPLC-MS chromatogram of dihydroferulate ion ([M-H]^-^*m/z* = 195.0657; calculated = 195.0657, 0 ppm error) with MS/MS spectrum inset. **B)** Ion mobility spectrum of dihydroferulate ion ([M-H]^-^). **C)** Selected ion UPLC-MS chromatogram of ferulate ion ([M-H]^-^*m/z* = 193.0500; calculated = 193.0500, 0 ppm error) with MS/MS spectrum inset. **D)** Ion mobility spectrum of ferulate ion ([M-H]^-^). **E)** 2-D plot of drift time vs. *m/z* for combined mixture of dihydroferulate and ferulate. Arrows indicate position of the two compounds. Image resolution was set to 1000 bins in the MS dimension to allow for ease of viewing of the ions.

Continuing with the overall trend of higher drift times based on larger mass, ferulate ([M-H]^-^*m/z* 193.0501), the 3-*O*-methylated derivative of caffeate, and its saturated derivative, dihydroferulate ([M-H]^-^*m/z* 195.0657), displayed the best separation among the hydroxycinnamate pairs (see Figure 3) when analyzed by either UPLC-MS or UPLC-IMS, with drift times of 2.14 ms and 2.34 ms, respectively. Being the largest of the hydroxycinnamates, these compounds displayed the lowest mobilities. Being more polar than *t*-cinnamate and dihydrocinnamate but less polar than caffeate/dihydrocaffeate, these compounds had intermediate retention times from the UPLC. As shown in Figure 3E, they were readily distinguishable and identifiable in the 2-D plots in Driftscope.

**Figure 4:**
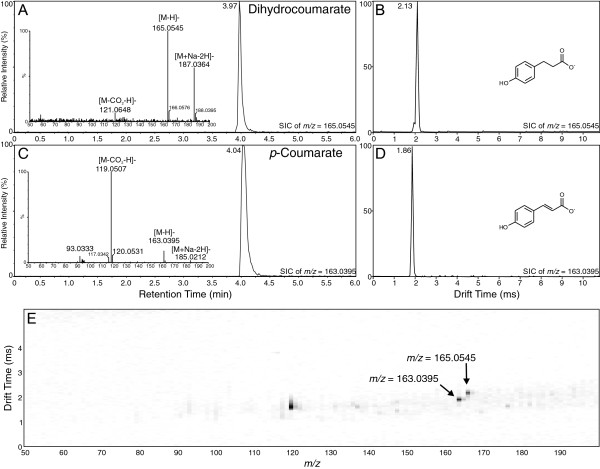
**Analysis of dihydrocoumarate and p-coumarate by UPLC-Q-IMS-TOFMS. A)** Selected ion UPLC-MS chromatogram of dihydrocoumarate ion ([M-H]^-^*m/z* = 165.0545; calculated = 165.0552, 4.24 ppm error) with MS/MS spectrum inset. **B)** Ion mobility spectrum of dihydrocoumarate ion ([M-H]^-^). **C)** Selected ion UPLC-MS chromatogram of *p*-coumarate ion ([M-H]^-^*m/z* = 163.0395; calculated = 163.0395, 0 ppm error) with MS/MS spectrum inset. **D)** Ion mobility spectrum of *p*-coumarate ion ([M-H]^-^). **E)** 2-D plot of drift time vs. *m/z* for combined mixture of dihydrocoumarate and *p*-coumarate. Arrows indicate position of the two compounds. Image resolution was set to 1000 bins in the MS dimension to allow for ease of viewing of the ions.

A more complex compound pair includes *p*-coumarate ([M-H]^-^*m/z* 163.0395) and *p*-dihydrocoumarate ([M-H]^-^*m/z* 165.0545), see Figure 4. Hydroxylation of the *t*-cinnamate phenyl ring at the *para*-position leads to *p*-coumarate, which is more polar (earlier elution from the UPLC column) than *t*-cinnamate and ferulate, but less polar than caffeate. As was observed for the *t*-cinnamate/dihydrocinnamate pair, the drift time of *p*-coumarate (1.86 ms) was shorter than that of *p*-dihydrocoumarate (2.13 ms). In addition, the drift time of *p*-dihydrocoumarate was longer than that of dihydrocinnamate, its non-hydroxylated but less massive analog. What was surprising was that the drift time of *p*-coumarate (1.86 ms) was lower than that of *t*-cinnamate (1.91 ms), a presumably more compact molecule, whereas the drift time of *p*-dihydrocoumarate (2.13 ms) was longer than that of dihydrocaffeate (see above). The cause of these differences may be explained by the nature of the phenolic hydroxyl groups of *p*-coumarate and *p*-dihydrocoumarate, but that is yet to be determined experimentally. Although *p*-coumarate and *p*-dihydrocoumarate were not readily separated in the UPLC-MS analysis, with overlapping chromatograms, they were readily separated, almost to baseline, in the ion mobility analysis (Figure 4B, D and E).

### Analysis of saturated and unsaturated chalcones and glycosylated derivatives

**Figure 5:**
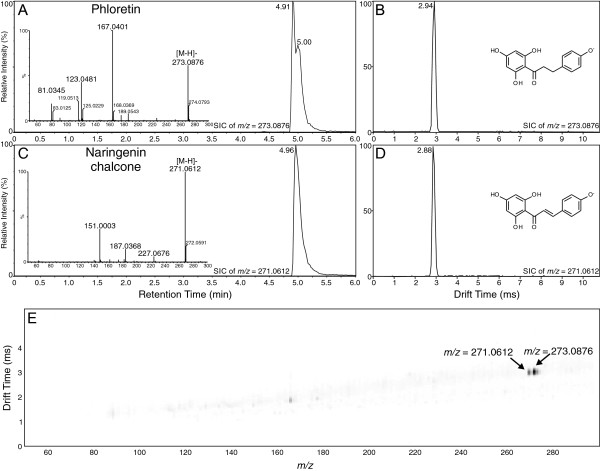
**Analysis of phloretin (dihydronaringenin chalcone) and naringenin chalcone by UPLC-Q-IMS-TOFMS. A)** Selected ion UPLC-MS chromatogram of phloretin ion ([M-H]^-^*m/z* = 273.0876; calculated = 273.0763, -41.38 ppm error) with MS/MS spectrum inset. **B)** Ion mobility spectrum of phloretin ion ([M-H]^-^). **C)** Selected ion UPLC-MS chromatogram of naringenin chalcone ion ([M-H]^-^*m/z* = 271.0612; calculated = 271.0607, 1.84 ppm error) with MS/MS spectrum inset. **D)** Ion mobility spectrum of naringenin chalcone ion ([M-H]^-^). **E)** 2-D plot of drift time vs. *m/z* for combined mixture of phloretin and naringenin chalcone. Arrows indicate position of the two compounds. Image resolution was set to 1000 bins in the MS dimension to allow for ease of viewing of the ions.

In addition to the hydroxycinnamates described above, two chalcones (naringenin chalcone and naringin chalcone) and their saturated derivatives (phloretin and dihydronaringin chalcone, respectively) were evaluated for their ability to be separated and analyzed by UPLC-MS and IMS under the generic non-targeted metabolomics-based conditions employed in this investigation. As shown in Figure 5, naringenin chalcone ([M-H]^-^*m/z* 271.0612) and phloretin ([M-H]^-^*m/z* 273.0876, the dihydro form of naringenin chalcone) essentially coeluted from the UPLC and had very similar and overlapping drift times in the IMS analysis. The 2-D plot from Driftscope (Figure 5E) shows the overlap in the IMS dimension very clearly. If a low resolution mass spectrometer were to be used in a non-targeted LC-MS/MS-based metabolomics experiment designed to analyze a plant or food extract that was expected to contain these two compounds, and phloretin were present at low levels, it would be impossible to tell it from the 2× ^13^C isotopomer of naringenin chalcone using MS analysis alone. Thus, the only way to be sure that both compounds are present is to analyze them by high resolution mass spectrometry or to use a triple-quadrupole instrument with an MRM (multireaction monitoring) approach. The latter instrument is typically not used in metabolomic investigations as it is ideally suited instead for targeted metabolite analysis. Thus, even though the data outlined above and other results reported elsewhere [16] showed that ion mobility can be a powerful tool for the analysis of biomolecules, it is not the approach to answer every question.

**Figure 6:**
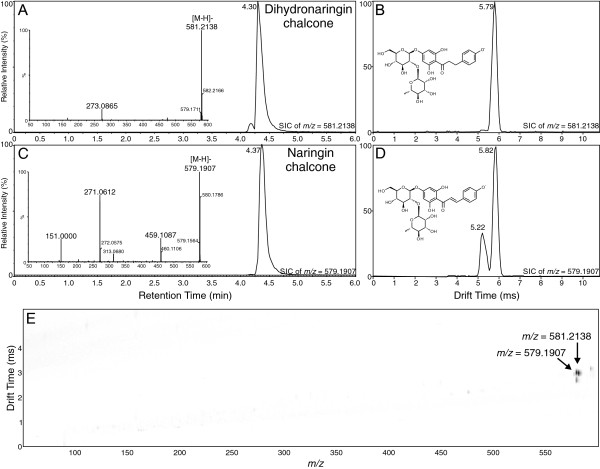
**Analysis of dihydronaringin chalcone and naringin chalcone by UPLC-Q-IMS-TOFMS. A)** Selected ion UPLC-MS chromatogram of dihydronaringin chalcone ion ([M-H]^-^*m/z* = 581.2138; calculated = 581.1870, -46.11 ppm error) with MS/MS spectrum inset. **B)** Ion mobility spectrum of dihydronaringin chalcone ion ([M-H]^-^). **C)** Selected ion UPLC-MS chromatogram of naringin chalcone ion ([M-H]^-^*m/z* = 579.1907; calculated = 579.1714, -33.32 ppm error) with MS/MS spectrum inset. **D)** Ion mobility spectrum of naringin chalcone ion ([M-H]^-^). **E)** 2-D plot of drift time vs. *m/z* for combined mixture of dihydronaringin chalcone and naringin chalcone. Arrows indicate position of the two compounds. Image resolution was set to 1000 bins in the MS dimension to allow for ease of viewing of the ions.

Naringin chalcone is a glycosylated derivative of naringenin chalcone, with glycosylation occurring on the A ring at position 4 (see Figure 6). As seen for several of the other compounds in this investigation, naringin chalcone ([M-H]^-^*m/z* 579.1907) and its saturated derivative (dihydronaringin chalcone, [M-H]^-^*m/z* 581.2138) shared essentially overlapping elution volumes in the UPLC-MS analysis and very similar behavior in the ion mobility analysis, with drift times of 5.79 ms for dihydronaringin chalcone and 5.82 ms for the major conformer of naringin chalcone. A second mobility peak with the same exact mass as the latter compound, with a drift time of 5.22 ms, was clearly visible in the 1D and 2D plots (Figure 6D and E). This peak likely represents a different isomer of naringin chalcone. In any case, the overlapping drift times and UPLC elutions for naringin chalcone and dihydronaringin chalcone suggest that, as for the narginenin chalcone/phloretin story, the only way to tell these two compounds apart in MS-based metabolomics investigations is via high resolution mass spectrometry. Nevertheless, use of IMS in the analysis leads to higher confidence in identification of these compounds, when mobility values are known.

### Experimental

#### *Chemicals*

Standards of several phenylpropanoid acids (*t*-cinnamate, dihydrocinnamate, *p*-coumarate, *p*-dihydrocoumarate, caffeate, dihydrocaffeate, ferulate, dihydroferulate), chalcones and dihydrochalcones, as well as acetonitrile, ammonium acetate, formic acid, and HPLC grade water, were purchased from Sigma-Aldrich (St. Louis, MO, USA). The other chalcones used in this investigation were synthesized and purified based on a previously published protocol [17]. All chemicals were dissolved in acetonitrile-water with 0.1% (v/v) formic acid, at a final concentration of 100 μM.

#### *Chromatographic separation*

Chromatography was carried out in an Acquity UPLC system using an Acquity UPLC BEH C18 column of 1.7 μm particle size (2.1 mm × 100 mm), both from Waters (Milford, MA, USA).

UPLC conditions were optimized in order to achieve good chromatographic resolution and sensitivity. Chromatography was carried out at 0.2 mL · min^-1^ (total solvent flow rate) and 30°C (column temperature). Different mobile phases and pHs ranging from 2–11 were tested. Finally, the mobile phase that provided the best resolution and which was used for all analyses described consisted of: solvent A, 0.1% formic acid and 5 mM ammonium formate in water; and solvent B, 100% acetonitrile and 0.1% formic acid. The linear gradient system used was 5% B in A (for 1.71 min), 5–65% B (2.29 min), 65-100% B (0.5 min), 100% B (0.5 min), 100-5% B (2 min). Compound elution was monitored at 200–400 nm with a Waters UPLC LG500 nm UV⁄Vis photodiode array detector. Sample injection volume was 5 μL.

#### *Mass spectrometry and ion mobility*

All measurements were performed on a SYNAPT™ G2 HMDS spectrometer (Waters, Milford, MA, USA), which is a hybrid quadrupole time-of-flight (Q-TOF) mass spectrometer with an inline traveling-wave ion mobility spectrometer (TWIMS) between the quadrupole and the TOF. This instrument was connected inline for all experiments with the Acquity system described just above. Thus, the outlet of the LG500 nm UV⁄Vis photodiode array detector was transferred directly to the inlet of the SYNAPT™ G2 HMDS, thereby enabling direct comparison of UPLC-UV/Vis analysis of specific analytes to Q-IMS-TOFMS analysis in the SYNAPT G2 HDMS system. Electrospray ionization negative mode (ESI-) was used with a capillary voltage of 3.0 kV. The ion source block and nitrogen desolvation gas temperatures were set to 80 and 100°C, respectively. Cone voltage was 25 V, desolvation gas temperature was 450°C and desolvation gas flow was 600 L · h^-1^. The Mobility T-Wave was operated with a velocity of 250 m · s^-1^, the pulse height optimized between 7 and 9 V and the helium pressure at 3.0 mbar. The TOF-MS spectra were obtained in “V” mode operating at a resolution of >10,000 full width at half maximum (FWHM). The mass range acquired was 50–1200 *m/z*. Data acquisition and processing were carried out using MassLynx (v. 4.1) and Driftscope (v. 2.1) software supplied with the instrument (Waters, Milford, MA, USA).

## Conclusions

In this investigation, ion mobility spectrometry was coupled to UPLC-MS and provided a rapid separation step that enabled a two-dimensional analysis in which chemical noise prevalent in mass spectrometry was significantly reduced (see panel E in Figures 1, 2, 3, 4, 5 and 6). As outlined for the phenylpropanoic acids, IMS also enabled rapid separations of very similar molecules that differed only by the presence or absence of a double bond. There will be cases where it is impossible to resolve such similar molecules by this technique, as was the case for the two chalcones and their dihydrochalcone derivatives. This was likely due to the very small size (collisional cross section area) differences compared to their overall larger masses, although specific conformation preferences for the molecules may have played a role as well. However, as ion mobility resolution and separation power increase, this technique may be able to resolve such compounds or other compounds of similar sizes that differ by such small amounts in mass and structure.

## Abbreviations

CSH: Charged surface hybrid; ESI: Electrospray ionization negative mode; FWHM: Full width at half maximum; HSS: High strength silica; IMS: Ion mobility spectrometry; MRM: Multireaction monitoring; Q-TOF: Quadrupole time-of-flight; TWIMS: Traveling-wave ion mobility spectrometer; UPLC-Q-IMS-TOFMS: ion mobility spectrometry coupled to UPLC-Q-TOFMS.

## Competing interests

The authors declare that they have no competing interests.

## Authors’ contributions

MI performed the experiments and drafted the manuscript. MI and DRG participated in the conception, design of study, revision of the manuscript, and approved the final manuscript.
